# Calvarial Suture-Derived Stem Cells and Their Contribution to Cranial Bone Repair

**DOI:** 10.3389/fphys.2017.00956

**Published:** 2017-11-27

**Authors:** Daniel H. Doro, Agamemnon E. Grigoriadis, Karen J. Liu

**Affiliations:** Centre for Craniofacial and Regenerative Biology, King's College London, Guy's Hospital, London, United Kingdom

**Keywords:** skull, calvaria, bone, stem cells, repair, cranial

## Abstract

In addition to the natural turnover during life, the bones in the skeleton possess the ability to self-repair in response to injury or disease-related bone loss. Based on studies of bone defect models, both processes are largely supported by resident stem cells. In the long bones, the source of skeletal stem cells has been widely investigated over the years, where the major stem cell population is thought to reside in the perivascular niche of the bone marrow. In contrast, we have very limited knowledge about the stem cells contributing to the repair of calvarial bones. In fact, until recently, the presence of specific stem cells in adult craniofacial bones was uncertain. These flat bones are mainly formed via intramembranous rather than endochondral ossification and thus contain minimal bone marrow space. It has been previously proposed that the overlying periosteum and underlying dura mater provide osteoprogenitors for calvarial bone repair. Nonetheless, recent studies have identified a major stem cell population within the suture mesenchyme with multiple differentiation abilities and intrinsic reparative potential. Here we provide an updated review of calvarial stem cells and potential mechanisms of regulation in the context of skull injury repair.

## Introduction: skeletal stem cells (SSCs)

Skeletal stem cells (SSCs) provide the bones with a supply of osteochondroprogenitors during development, modeling and life-long homeostasis (Park et al., 2012). More importantly, these cells are crucial for appropriate self-healing in response to injury or general bone loss. Bone marrow (BM) residing stem cells have been suggested to assist the repair by establishing a pro-regenerative hematopoietic microenvironment, but also by supplying progenitors for all the newly formed skeletal components (Grcevic et al., 2012; Park et al., 2012).

**Figure 1:**
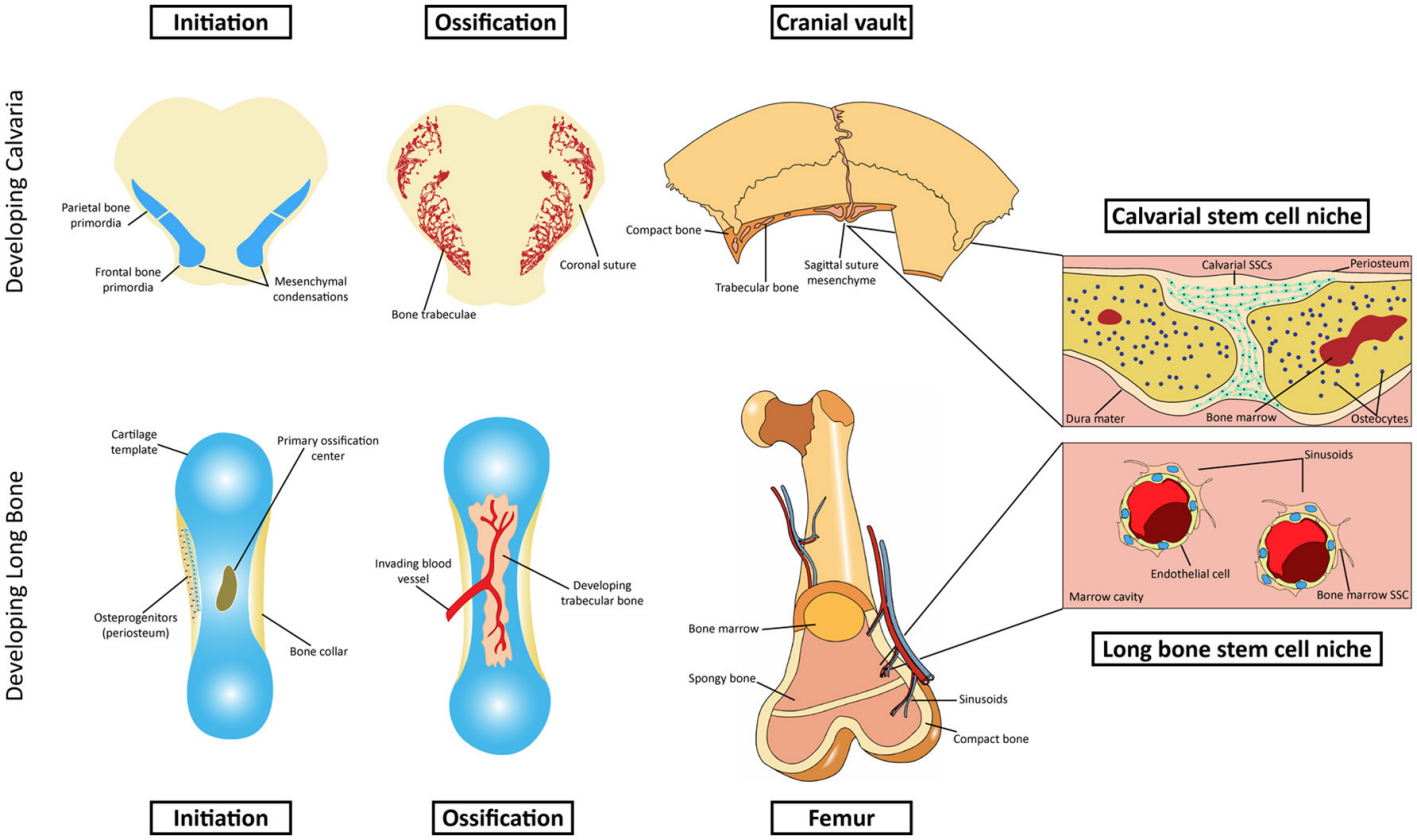
Schematic representation of intramembranous and endochondral ossification. Top row: Initiation and development of the intramembranous calvarial bone. The calvarial stem cell resides primarily in the sutural mesenchyme. Bottom row: Initiation and development of endochondral bone. The stem cell niche in endochondral bone is more complex, residing in the marrow cavity.

Defining specific stem cell subsets and their ontogeny within the stromal system remains a challenge, as the discovery of numerous markers and isolation methods reveals a large phenotypic heterogeneity of the SSC population (Bianco and Robey, 2015). Fundamental studies leading to the initial characterization of SSCs were based on heterotopic transplantation of bone marrow cell suspensions or bone-devoid BM explants into extramedullary sites. Both populations were able to generate an ectopic ossicle, similar in tissue composition and architecture to trabecular bone and its stroma, fulfilling the first of two essential criteria that define a stem cell population—the *in vivo* multipotency (Friedenstein et al., 1968; Tavassoli and Crosby, 1968). The second criterion, self-renewal capacity, was met later as isolated clonogenic cells were found able to reconstitute progenitors with identical phenotype, potency and equivalent anatomical location within the ectopic bony structure formed (Friedenstein et al., 1974; Kuznetsov et al., 1997). Further knowledge about phenotypic identity and anatomical location of SSCs has been accumulated upon discovery of cell-surface markers like human-CD146 (Sacchetti et al., 2007) and mouse-CD45/CD200 (Chan et al., 2015), or even proteins expressed by specific SSC subpopulations like NESTIN (Méndez-Ferrer et al., 2010) and GREM1 (Worthley et al., 2015). Taken together, these studies agree on the widely reported perivascular niche of SSCs in the long bone marrow (Figure 1). Cells labeled with the CD146 marker are found in the adventitial layer of sinusoidal walls with typical reticular morphology, reminiscent of previously described ARCs (adventitial reticular cells) (Sacchetti et al., 2007).

Across studies, however, the distinctively identified populations vary in their multipotency, as some of them can generate *in vivo* osteoblasts, chondrocytes and stromal cells, but not adipocytes (Worthley et al., 2015). Rather than conflicting findings, these results point toward phenotypic heterogeneity of the SSCs, revealing that what were once generically called bone marrow mesenchymal stem cells actually comprise a number of subsets with subtly different properties. Many aspects regarding ontogeny and regulatory networks in long bone stromal SSCs have yet to be elucidated. Nevertheless, the knowledge accumulated over the years far exceeds that of stem cell populations serving the calvarial bones, which only recently started to be characterized in detail. Our aim here is to provide a comprehensive summary of recently reported stem cell populations within the cranial suture mesenchyme, their roles during postnatal turnover and wound repair and proposed mechanisms of regulation.

## Long bone SSCs: where do they come from?

Embryonic development of the long bones occurs via endochondral ossification and begins with the condensation of mesenchymal cells derived from lateral plate or paraxial mesoderm (reviewed in Olsen et al., 2000). These cells differentiate into chondrocytes and lay down a cartilaginous anlagen, which is later replaced by the osteoblasts and bone formation (see Figure 1). Chondrocytes in the center enlarge to a hypertrophic state allowing bone growth and inducing ossification in the peripheral layer of undifferentiated cells around the cartilage, the perichondrium (Kronenberg, 2003). Secretion of vascular endothelial growth factor (VEGF) attracts blood vessels and chondroclasts, cells specialized in digesting the cartilage matrix, which will then invade the ossified bone collar (Arai et al., 2002). Upon apoptosis of the hypertrophic chondrocytes, the hollow cavities left in the matrix are invaded by blood vessels and osteoblasts, which will lay down bone matrix on top of a calcified cartilage scaffold to form primary spongy or trabecular bone. Once the marrow space is established, the bone is finally invaded by stromal osteoprogenitors, some of which are recruited to the perivascular niche of large caliber sinusoids where they will reside as bone marrow skeletal stem cells. Prior to invasion and formation of bone marrow stroma, these cells are reportedly derived from proliferating perichondrial precursors. Phenotypic similarity of cells in the perichondrium with primitive stromal cells was observed in early studies (Bianco et al., 1993). Their origin was later confirmed by immunostaining of the perichondrial marker ALCAM (activated leukocyte cell adhesion molecule) (ALCAM). FAC-sorted ALCAM positive cells showed characteristics of mesenchymal stem cells and ability to differentiate into all skeletal lineages (Arai et al., 2002). Moreover, osterix expressing perichondrial/periosteal cells, labeled prior to vascular invasion, give rise to trabecular osteoblasts, osteocytes and stromal cells inside the developing bone, confirming the unequivocal origin of skeletal stem cells in the undifferentiated perichondrium (Maes et al., 2010; Staines et al., 2013).

## Anatomical and developmental differences point to distinct stem cell niche in calvarial bones

In contrast with the extensively studied stem cell niche in the endochondral bones, the source of SSCs in cranial bones was, until recent years, neglected or generally assumed to be identical to the long bones. Given the limited bone marrow space in these bones and very distinct developmental history, it is unsurprising that the local stem cell population would reside in a different compartment (Figure 1). The majority of craniofacial bones derive from the neural crest, with a few exceptions like mesodermally-derived parietal bones (Jiang et al., 2002). The latter will compose, together with crest-derived frontal bones, the top part of the skull called the cranial vault, or calvaria, which is fully made via intramembranous ossification. Although early mesenchymal condensations are also required in membranous bone formation (Hall and Miyake, 2000), these cells do not assume a chondrogenic fate or lay down cartilaginous scaffolds as in endochondral bones. Rather, upon expansion of the condensed mesenchyme, the cells in the center differentiate directly into osteoblasts, which will then secrete unossified matrix (osteoid) that will be later mineralized into compact bone. Growth of the immature bone then occurs at the leading edges of separate bones, dependent on proliferation and subsequent differentiation of osteoprogenitors at the commonly called osteogenic front (Rice et al., 2003). As the osteogenic fronts of two bones approach each other, they are interposed by undifferentiated mesenchyme, forming fibrous joints called sutures. Throughout embryonic and postnatal development, the sutures remain as an active site of bone formation in the expanding skull. The studies of Lana-Elola et al. (2007) helped to elucidate the mechanisms of bone growth and the contribution of different lineages within the suture environment. Using vital dye labeling, it was found that the primary mechanism for parietal bone growth is via proliferation and differentiation of leading edge osteoprogenitors with a surprisingly minor contribution of sutural mesenchymal cells, provided they are adjacent to the osteogenic fronts (Lana-Elola et al., 2007). Growth at the suture is also finely controlled by signaling from the underlying dura mater (Kim et al., 1998). Absence of dura mater leads to osseous obliteration of the coronal suture, arresting the active growth at that site (Opperman et al., 1995). Moreover, the orientation of the dura mater under the cranial vault is critical for temporal control over suture patency or fusion (Levine et al., 1998). Growth and remodeling of the skull is also influenced by the overlying periosteum, a stratified membrane of mesenchymal cells including fibroblasts and osteoprogenitors (Brey et al., 2007). Mechanisms of periosteal activity during appositional growth and bone resorption of the cranial vault remain unclear. However, the membrane has been suggested to regulate osteogenesis via paracrine signaling (Cadet et al., 2003) and to provide osteoprogenitors that support craniofacial repair (Ochareon and Herring, 2011). Unlike the thoroughly described bone marrow-residing SSCs as the main source of osteoprogenitors during long bone repair (Zhou et al., 2014), the contribution of calvarial specific stem cells for cranial regeneration was unknown until very recently. Although a clonal population with multipotent MSC differentiation properties has long been isolated from rat calvaria (Grigoriadis et al., 1988), the visual evidence of the niche where the calvarial SSCs reside was only provided in the last few years.

## SSCs residing in the cranial suture are the major contributors to injury repair

In the same way as BM-SSCs, calvarial stem cell research was largely propelled by the identification of specific markers expressed by resident populations. Only in the last 2 years, three populations were identified within the sutural mesenchyme and proposed as major calvarial skeletal stem cells, or subsets of it. Namely, *Gli1* positive (*Gli1*+), *Axin2*-expressing (*Axin2*+) and postnatal *Prx1*-expressing (*Prx1*+) cells, as per definition in the original references (Zhao et al., 2015; Maruyama et al., 2016; Wilk et al., 2017). The authors used similar approaches to characterize the suspected SSC population with regards to location, stem cell-like properties and other features shared by the cells described which we attempt to summarize in this section.

### Finding a good calvarial stem cell marker

Zhao et al. (2015) hypothesized that *Gli1* expressing cells were the MSCs for craniofacial bones, as they were for the incisor mesenchyme they reported earlier (Zhao et al., 2014). The zinc finger protein GLI1 is a well-known transcriptional effector of Hedgehog signaling (Hooper and Scott, 2005). Seidel et al. (2010) had previously proposed it as a dental stem cell marker upon discovery of Hh responsiveness (*Gli1*^*lacZ*^) from slow cycling cells of the dental epithelium (Seidel et al., 2010). The stem cell identity of these cells was later confirmed in the studies of Zhao et al. (2014). The AXIN2 protein, a negative regulator of Wnt signaling, was previously implicated in calvarial morphogenesis with *Axin2* knockout mice showing craniosynostosis (Yu et al., 2005). Maruyama et al. (2011) described the essential role of AXIN2 in orchestrating the signaling network (Wnt, BMP, FGF) that regulates mesenchymal cell fate determination (Maruyama et al., 2011). The protein was recently proposed as a calvarial stem cell marker as *Axin2*-expressing cells in the midline of the suture mesenchyme were found slow-cycling in nature (Maruyama et al., 2016). Finally, the transcription factor *Prx1* (also referred to as *Prrx1*) was previously shown to be highly expressed during limb bud formation and craniofacial development (Martin and Olson, 2000). Interestingly, PRX1 expression seemed to avert differentiation of early progenitors into committed osteoblasts, suggesting that the transcription factor could potentially mark SSCs in the calvarial bones (Lu et al., 2011). This hypothesis was successfully tested in the latest of the above-mentioned studies by Wilk et al. (2017).

### Distribution within the cranial mesenchyme

*Gli1*+ cells of the cranium are found in the entire periosteum, dura and suture mesenchyme at birth. However, from postnatal day 21 onwards these cells are gradually restricted to the cranial sutures where they remain throughout adulthood (Zhao et al., 2015). *Axin2* was previously found to be expressed in the midline of sagittal suture skeletogenic mesenchyme. In a more recent study, *Axin2* expression was reported in all calvarial sutures, co-localizing with slow cycling cells that retained EdU (Maruyama et al., 2016). Unlike the *Gli1*+ cells, no obvious mention or visual evidence of early expression in the dura or periosteum is seen. In fact, *Axin2* expression is reportedly restricted to the midline of the sutural mesenchyme from postnatal day 10, whereas *Gli1*+ tracing seems to target a larger area within the suture. Wilk et al. (2017) investigated postnatal *Prx1*+ cells and found them exclusively in the calvarial sutures from 2 weeks of age until late adulthood. Like *Axin2*-expressing cells, *Prx1*+ were also not found in the periosteum or dura mater, although no mention or visual evidence of early postnatal expression is given (as in Zhao et al., 2015). Overall, the markers described seem to identify cells in a common niche. Whether the populations overlap in identity and function, or represent distinct functional subsets of a main stem cell source is still under debate as further discussed below. No expression of the above mentioned markers was detected in the posterior frontal suture, which likely correlates with the fact that this is the only calvarial suture fused at that stage in mouse development.

### Self-renewal and ability to generate all calvarial tissues

As previously mentioned in this review, self-renewal ability and *in vivo* multipotency are the cardinal requirements of a *bone fide* stem cell. While the *in vivo* differentiation potential can be assessed in many ways, some authors argue that self-renewal should only be assessed via heterotopic transplantation of non-induced cultures (Bianco and Robey, 2015). A typical SSC after being cultured and clonally isolated should still be able to generate all the skeletal compartments (multipotency) and “a cell compartment anatomically, phenotypically and functionally equivalent to the one originally explanted” (self-renewal) (Bianco and Robey, 2015). This remains the mainstay of MSC investigation criteria, as it honors the concept of stem cell autonomy in making, without the need for any artificial cues, organized skeletal tissue, including a reservoir of stem cells. In the case of calvarial stem cells, this would mean the ability to make *in vivo*, after culture and clonal isolation, all the components of a membranous bone and a mesenchymal equivalent to the cranial sutures.

Although heterotopic transplantation of putative calvarial SSCs was not the approach taken, the evidence presented in the studies reviewed herein strongly supports the ability of the putative stem cells to generate all calvarial compartments *in vivo*. Firstly, single pulse labeling (induced by tamoxifen in *Gli1-CreERT2* and *Prx1-creER* mice, or doxycycline in *Axin2-cre-Dox*) provides that only the cells initially labeled within the sutural mesenchyme and their progeny are being tracked (Zhao et al., 2015; Maruyama et al., 2016; Wilk et al., 2017). In all studies, the co-expression of osteogenic markers like runx2, osterix, collagen type 1, osteocalcin and sclerostin was not observed in the labeled suture mesenchyme, confirming that the proposed stem cell populations were undoubtedly distinct from any postnatal preosteoblasts and mature osteoblasts. *Gli1*+ cells were permanently labeled at 1 month of age and found increasingly abundant in the suture mesenchyme, periosteum, dura mater and parts of the calvarial bones up to 8 months of age (Zhao et al., 2015). In a similar manner, derivatives of the *Axin2*+ cells permanently labeled at 1 month, accumulated continuously in the sagittal suture until after 1 year of development, with a small number of cells found embedded in the bone matrix near the osteogenic fronts. However, this study did not report any contributions of the cells in question to the mature bone and surrounding tissues (dura mater and periosteum). Conversely, a large contribution of *Prx1*+ cells to all calvarial tissues, except bone marrow osteoblasts, was observed in 6–8 week old mice (Wilk et al., 2017). *Prx1* expression is detected as early as embryonic day 15, if not earlier, in the developing skull (Ouyang et al., 2013). Unfortunately, the conclusions about postnatal *Prx1*+ contribution to adult tissues were drawn from constitutive *Prx1-cre* lineage tracing analysis. This could include, although unlikely, cells that were specified immediately before the formation, or turn-over of the calvarial components analyzed, without definitive proof that they came from the sutural niche. Moreover, although Zhao and Wilk make no specific claims about self-renewal properties of the stem cell populations reported, Maruyama goes so far as to say that the accumulation of *Axin2*+ cells within the 1 year period reflects a self-renewal capability of those cells (Maruyama et al., 2016); however, this could also be due to a high proliferation capacity (Bianco and Robey, 2015). Nonetheless, Maruyama provides rigorous and more compelling evidence of *in vivo* stem cell behavior by transplanting isolated multi-colored (*R26R*^*Confetti*^) *Axin2*^+^ cells to an extra-skeletal site (kidney capsule), which the other authors did not attempt. Ectopic bones labeled by single fluorescent colors were observed 3 weeks after transplantation, indicating that these cells can clonally expand *in vivo* and differentiate autonomously into the osteogenic lineage (Maruyama et al., 2016). However, contrary to bone marrow residing skeletal stem cells, *Axin2*^+^ SCs from the suture were not able to generate cartilage, unless artificially stimulated by BMP2, which drifts away from the principle of non-cued multipotency toward skeletal lineages expected from *bona fide* SSCs. While this could be a specific feature of membranous bone residing stem cells, ectopic chondrogenesis has been previously reported in the sutural mesenchyme, suggesting that they possess the intrinsic ability to differentiate into the chondrogenic lineage (Grigoriadis et al., 1988; Maruyama et al., 2011).

### MSC *in vitro*/*ex vivo* behavior

Aside from rigorously established criteria, MSCs are popularly defined by their *in vitro* culture properties, including the ability to differentiate into various cell types (osteoblasts, chondrocytes, adipocytes and, arguably, neurons) (Keating, 2012). Likewise, although with considerable disagreement within the stem cell field, MSCs are loosely defined based on the expression profile of numerous surface markers (Dominici et al., 2006). Typical MSC markers CD90, CD73, CD44, *Sca1*, and CD146 were not detected in the majority of *Gli1*^+^ cells *in vivo*. However, after 1 week of culture, fluorescent-activated *Gli1*^+^ sorted cells express high levels of such MSC markers. *Gli1*^+^ cells are also clonogenic *in vitro* and subcultured clones were successfully differentiated into osteo-, chondro-, and adipogenic lineages under appropriate conditions (Zhao et al., 2015). With the exception of the recently reported Leptin receptor (Zhou et al., 2014), none of the bone marrow SSC markers investigated by Maruyama was distinctively expressed in the *Axin2*^+^ cells of the suture (*Mcam/CD146, Nestin and Gremlin1*). Moreover, the *in vitro* differentiation ability of these cells was not assessed in this particular study. *Axin2*^+^ isolated cells were, nevertheless, able to form colonies *in vitro* and to differentiate, after heterotopic transplantation, into the osteogenic lineage and into chondrogenic, although only upon external stimulation (BMP2), as previously mentioned (Maruyama et al., 2016). Postnatal *Prx1*^+^ cells are also clonogenic and exhibit a SSC-like profile *ex vivo*, including: upregulation of SSC markers *Pdgfr*α and *Mcam/CD146;* downregulation of cell cycle and DNA replication markers *Ccne2, Mcm4*, and *Pcna*—typical of quiescent cells; and high levels of transcripts associated with stem cell homing (*Itga2, Itga3*, and *Itga6*). Upon *in vitro* and *in vivo* stimulation with recombinant WNT3A, these cells are pushed toward osteogenic differentiation, judging by upregulated *Osx* and *Col1* and reduced expression of chondrogenic markers *Sox9* and *Col2* (Wilk et al., 2017). Altogether, the variable approaches, contrasting profiles and requirement of cueing factors for multi-differentiation in these studies points to the unreliability of loosely defined criteria to identify SSCs, based on which virtually every tissue can provide “stem cell” equivalents.

### Contribution to repair

The notion that skeletal stem cells work as postnatal contributors for tissue regeneration represents not only a general understanding of the organ physiology, but the very motivation for cautious and rigorous investigation of the stem cell populations, aiming at their translational potential. Although classically established requirements for SSCs do not include their regenerative properties, more than it does their multi-lineage differentiation ability in the context of organogenesis and postnatal turn-over, investigating the role of these cells during injury repair is essential, as the pursuit for stem cell-based therapies demand such knowledge. Therefore, the mechanisms of SSC recruitment and contribution to calvarial wound repair should accompany the research of any reported populations, as the three current studies highlighted here have done well to address. *Gli1*^+^ cells respond immediately to injury in the suture by rapidly activating proliferation within 24 h post incision at the sagittal midline. Contribution of *Gli1*^+^ cells to calvarial repair is observed in the vast infiltration of conditionally labeled cells into a 1 mm wide parietal wound situated 2 mm away from the sagittal suture. Two weeks after the injury is made, the majority of cells in the area are strongly labeled and, at 1 month, the repaired bone is mostly composed of *Gli1*^+^ osteocytes as well as positively labeled periosteum and dura mater. Orthotopic transplantation of a lineage traced calvarial bone piece containing sagittal suture into a 4mm defect, revealed a significant number of labeled cells within 1 month-regenerated periosteum, dura mater and bone, whereas an explant lacking the suture failed to generate any of these components. Even with intact dura mater and periosteum, calvarial explants devoid of suture were incapable of regenerating the wounded bone in the recipient mice. The results indicate that, contrary to the previous idea that progenitors involved in cranial repair mainly resided in the periosteum, the sutural mesenchyme and not the surrounding membranes possesses the regenerative capacity (Zhao et al., 2015). Similarly to the *Gli1*+ population, *Axin2*+ lineage traced cells here drastically expanded in the sutural mesenchyme in response to a 1.4 mm parietal bone defect. Four weeks after the surgical procedure, large infiltration of the injury site by *Axin2*^+^ cells is observed and co-localization with OSX (osteoprogenitors) and SOST (osteocytes) expression strongly indicates direct contribution to bone repair. *Axin2*^+^ cells were also shown to improve injury repair when directly transplanted into the wound site. While *Axin2* negative cells from the suture mesenchyme provide no better healing than that seen in non-transplanted injuries, *Axin2*^+^ cell transplantation significantly increased the repaired area 2 and 4 weeks after surgery. As in the previous experiment, the lineage labeling overlapped with markers of osteoprogenitors and osteocytes, confirming direct contribution of the *Axin2*+ population to repair of the wounded area (Maruyama et al., 2016).

Finally, *Prx1*^+^ cells actively contributed to regeneration of subcritical size defects in both frontal and parietal bones, although in this study a smaller 100 μm wide injury was made. Nevertheless, lineage traced *Prx1*^+^ cells were increasingly present in the wound area 5, 10, and 30 days post-surgery, when they co-localized to osteoblasts and osteocytes embedded in the newly formed bone. Moreover, the regeneration of, questionably, critical-sized (2 mm) defects was greatly improved by heterotopic transplantation of minced sutures into the wound, where *Prx1* labeling was seen in the majority of cells integrating the repaired bone 4 weeks after surgery. Interestingly, the repair of parietal subcritical defect did not occur in suturectomized mice after 4 weeks, whereas the 0.5 mm wound is fully repaired in mice with intact sagittal and coronal sutures (Wilk et al., 2017). Taken together, these studies confirm the unequivocal and potentially exclusive contribution of the sutural mesenchyme to calvarial injury repair.

### Requirement for development, homeostasis and injury repair

Overall, the studies described provide compelling evidence for direct (by providing osteoprogenitors) as well as indirect (via paracrine signaling) contribution of the putative stem cell populations to calvarial repair. Predominant infiltration of *Gli1*^+^*/Axin2*^+^*/Prx1*^+^ cells is seen in all the subcritical size defect assays, whereby the healing process should be accomplished by natural mechanisms occurring during normal bone physiology. Moreover, transplantation of these cells to the wound site showed significant repair improvement in all the attempts described in the previous section, supporting the translational potential of calvarial SSCs in a number of clinical scenarios. In two of the studies, notwithstanding, the authors found it appropriate to investigate whether the stem cell population described was indispensable for skull development, homeostasis and injury repair. While this is not a particular requirement for determining a resident stem cell, the findings provide valuable information on the ontogeny of the sutural niche and other calvarial compartments, as well as potential mechanisms of congenital disorders like craniosynostosis. Conditional ablation of *Gli1*^+^ *cells*, for instance, led to severe craniofacial phenotypes in *Gli1-Cre*^*ERT*2^*; DTA*^*flox*/*flox*^ mice (diphtheria toxin A). When ablation was induced at 1 month of age, fusion of ordinarily patent coronal and frontal-premaxilla sutures was observed after 1 month, leading to general reduction of DTA-mice body size at 2 months post-induction. At this stage, all the craniofacial sutures were fused and the bones of *Gli1*^+^ ablated mice presented severe osteoporosis. Repair of 1mm parietal defects was also compromised in DTA mice when compared to fully regenerated controls. Altogether, the results indicate that *Gli1*^+^ cells are largely required for craniofacial bone turnover and injury healing. Moreover, pathological fusion of the cranial sutures in *Gli1-*ablated mice suggests that a deficient supply of undifferentiated mesenchymal cells hinders the patency expected in calvarial sutures of adult mice (Zhao et al., 2015).

Two different strategies confirmed *Prx1*^+^ SSCs requirement for injury repair. Firstly, 8 week-old *Prx1-cre*^*ER*^; *Rosa26*^*DTA*/+^ mice were used to perform global ablation of *Prx1*^+^ cells during 5 days before and 5 days after 0.5 mm parietal wound surgery. Twenty 8 days later, micro-computed tomography and histological analysis showed drastic healing impairment in *Prx1*^+^ ablated mice against complete regeneration in the controls. Even in mice injured 2 months after ablation, parietal defects failed to regenerate as non-ablated mice. Secondly, excision of sagittal and right coronal sutures yielded non-regenerated wound in the right parietal bone, whereas removal of the opposing sutures (frontal and left coronal) did not affect the healing of parietal subcritical defect. This strongly suggests that calvarial bones depend on the SSCs of adjacent sutures to perform injury repair. Unlike *Gli1*^+^ SSCs, however, global ablation of postnatal *Prx1*^+^ cells did not result in craniosynostosis or any other major craniofacial phenotype. Indeed, *Gli1* expression is thought to identify a broadly distributed population of stem cells in the suture mesenchyme, of which *Prx1*+ cells constitute a subset seemingly dispensable for postnatal development and turnover. Ablation of embryonic *Prx1*+ cells leads to incomplete calvarial bone formation, suggesting that this specific subpopulation might only be required in earlier stages of development (Wilk et al., 2017). Whereas Maruyama did not perform any *Axin2*^+^ SSC ablation studies, *Axin2*-null mice are known to exhibit premature fusion of the cranial sutures (Yu et al., 2005), although this does not necessarily correlate with any particular requirement of the population concerned. Reduction of *Axin2*-expressing cells is seen upon ossification of the frontal suture (Maruyama et al., 2016). However, this is likely an effect on the entire undifferentiated mesenchyme at the interfrontal suture as it commits to the osteogenic lineage and promotes the fusion of the osteogenic fronts.

### Aspects of regulatory signaling

Throughout the skeleton, as a local niche is established, mesenchymal stem cells are subject to recruitment, proliferation, induction of mitotic quiescence and lineage commitment. Over the years, extensive research of bone marrow SSCs has shown that these steps are regulated by largely convoluted signaling pathways including: platelet-derived growth factor (PDGF) during perivascular recruitment (Sacchetti et al., 2007); parathyroid hormone (PTH) during expansion of the bone marrow stroma (Kuznetsov et al., 2004); TGF-β1 as the cells are maintained in a quiescent state (reviewed in Pepper, 1997; Jain, 2003) and extensive crosstalk between TGFβ/BMP, Wnt/β-catenin, FGF and Hedgehog pathways, which generally control MSCs lineage fate decision (reviewed in Cook and Genever, 2013). Given the complexity of the network necessary for developing and maintaining a stem cell niche, it is no surprise that regulatory signaling receives little attention in recent papers that attempt to describe calvarial SSCs. In fact, most of the preliminary evidence regarding regulation of calvarial SSCs arose from *in vitro* or unconvincing *in vivo* assays that were based on mechanisms previously established for stem cells in the long bones. Zhao et al. set out to investigate whether *Gli1*^+^ cells were, like BM-SSCs, regulated by hedgehog signaling in the context of lineage commitment. No Sonic hedgehog (Shh) signal was detected in the suture region using a R26Tdtomato reporter. However, Indian hedgehog (Ihh), a regulator of skeletal development, was found in cells of the osteogenic fronts flanking the cranial sutures. Deletion of the hedgehog receptor Smoothened (*Smo*) in *Gli1*^+^ cells did not affect suture patency, proliferation of *Gli1*^+^ cells, or their ability to generate periosteum, dura and osteocytes; nor did it induce apoptosis in the sagittal suture. Nonetheless, all the craniofacial bones in *SmoKO*^*Gli1*−*cre*^ mice exhibited severe osteoporosis and reduced bone volume after 8 months. *In vitro* analysis of sutural mesenchyme cells revealed up-regulated *Gli1* expression, increased osteogenic activity and upregulation of osteodifferentiation markers upon IHH treatment, whereas the hedgehog inhibitor GDC0449 induced the opposite effects. Neither treatment affected proliferation or apoptosis of cultured mesenchymal cells, suggesting that Hedgehog signaling might be important for regulation of *Gli1*^+^ SSC differentiation, but not their maintenance (Zhao et al., 2015). Maruyama and Wilk's insights on regulation of *Axin2*^+^ and *Prx1*+ cells, respectively, are merely suggestive, as these cells present *in vivo* expression of canonical Wnt inhibitors in their quiescent state and lineage fate decision changes upon exogenous activation of Wnt or BMP signaling. Transplanted *Axin2*^+^ cells were able to generate ectopic mineralized bone, but no cartilage after 7 days, contrasting the multi-lineage potential of long bone marrow SSCs. However, exogenous stimulation with BMP2 shifted *Axin2*^+^ cells from an osteogenic to a chondrogenic fate, shown by detection of Alcian blue (cartilage), but not von Kossa (bone) staining in the structure generated (Maruyama et al., 2016). Expression of Wnt inhibitors *Dkk1* and *Sost* by *Prx1*^+^ sorted cells supports the well-established idea that skeletal progenitors are maintained in an undifferentiated status, or yield chondrogenic differentiation in the absence of canonical Wnt activation (Day et al., 2005; Hill et al., 2005). Indeed, *in vivo* expression of chondrogenic markers *Sox9* and *Col2* was diminished in pnPRX1^+^ cells upon subperiosteal injection of Wnt agonist WNT3A to the cranial sutures, whereas osteogenic markers Osx and Col1 were significantly upregulated. Commitment to the osteogenic fate has also observed when these cells were treated in culture with recombinant WNT3A, endorsing the previously described role of Wnt/β-catenin pathway in differentiation of mesenchymal progenitors (Wilk et al., 2017). Altogether, recently published studies provide informative, yet inconclusive findings to characterize molecular regulation of calvarial SSCs, leaving exciting open prospects for future stem cell research.

## Discussion

As once wisely stated, “All definitions of stem cells are functional in nature, and all types of stem cells are defined by functional assays” (Bianco et al., 2013). That is, one must see that hasty and careless attempts to identify stem cells in the adult body will not prompt us to replace rigorously established criteria with simple surface marker profiling, or loosely defined *in vitro* properties. From the very concept of stemness, proposing a stem cell candidate requires that a single cell within the niche described can generate multiple and fully differentiated tissues *in vivo* and produce a reservoir of cells with identical phenotype and multipotency. At the risk of raising unrealistic expectations for the clinical use of stem cells, many authors over the years claimed to have isolated post-natal fibroblastic populations from virtually every tissue with differentiation abilities that extrapolate the tissue-specific potential of the identified progenitor, much like an embryonic stem cell (da Silva Meirelles et al., 2006). This apparent “pluripotency” attributed to putative post-natal stem cells is often based on chemically induced *in vitro* differentiation studies (Robey et al., 2014), which more accurately describe the artificial reprogramming of a cell than its *in vivo* potency. The very use of the term mesenchymal stem cell (MSC) to describe bone marrow stromal progenitors is misleading in that it is based on the erroneous and unproven hypothesis that stem cells in the bone marrow could generate various differentiated tissues beyond the skeletal lineages. It also assumes that MSCs throughout the body have a common developmental origin (Caplan, 1991) and can be found in a number of different organs sharing the same differentiation potential as BMSCs and, as previously hypothesized, equivalent perivascular niche (Chen et al., 2012). In reality, evidence originating from more stringent *in vivo* assays indicates that the bone marrow harbors stem cells exclusively for skeletal-tissues, namely, bone, cartilage, adipose tissue and stroma (Owen and Friedenstein, 1988; Sacchetti et al., 2007). Therefore, as previously proposed (Bianco et al., 2008), “skeletal stem cell” is a more adequate designation, for it describes the real potential of the so called bone marrow stromal cells and implies that stem cell populations identified in other tissues are not expected to behave exactly like SSCs in the long bones. In fact, the concept of tissue-specific stem cells as opposed to a homogeneous MSC population with multiple locations, accords with a more reasonable prediction that most adult tissues would maintain a stock of undifferentiated yet committed progenitors to support physiological turn-over and regeneration in response to injury.

The search for a calvarial stem cell then must indeed be grounded on very stringent criteria. However, the assessment of *in vivo* multipotency and self-renewal in this population need not be guided inflexibly by the sequential assays classically employed for BM-SSCs investigation (Bianco et al., 2013; Bianco and Robey, 2015), lest one must conclude that: either the membranous bones in the skull lack a resident stem cell population, or the well supported contribution of sutural mesenchymal progenitors to all calvarial components reflects a distinct role/mechanism of skeletal turn-over and injury repair, even though the same is performed by bone marrow SSCs in the long bones. For instance, it is fair to require that *in vivo* multipotency is assessed exclusively by heterotopic transplantation of clonally isolated calvarial cells rather than alternate *in vitro* assays. But, to expect the formation of an ectopic ossicle with all the compartments of an endochondral bone, contradicts the idea of organ-specific stem cells and resonates with the old farfetched concept of “pluripotent” MSCs. Moreover, the ability to self-renew is easily observed in ectopically transplanted BM-SSCs, as these cells generate organized bone marrow tissue and reconstitute a pool of identical undifferentiated progenitors (Sacchetti et al., 2007). However, since the stem cells committed to an intramembranous fate will not generate the compartments produced during an endochondral ossification, a different readout must be required as proof of self-renewal ability. Maruyama has shown that ectopically transplanted *Axin2*^+^ sutural SCs can clonally expand *in vivo*, but also generate bony structures, judging by monochromatic expansion of *R26-confetti* traced cells (Maruyama et al., 2016). Whether this could be taken as convincing evidence of stemness is still debatable, although it is surely more rigorous than otherwise defined *in vitro* MSC criteria (Dominici et al., 2006). Also, lineage-tracing approaches like the ones employed for the calvarial stem cell niche investigation might not be the mainstay for assessing *bona fide* stem cell criteria. Nonetheless, the conclusions drawn with regards to multi-lineage differentiation ability and injury repair contribution *in vivo* are well supported with single-pulse conditional labeling methods.

In summary, the stringent criteria defended by “rigorous *in vivo* assay” apologists stems from a genuine concern about negligent clinical application of poorly characterized stem cell candidates. Experienced stem cell scholars report the uprising of unrealistic expectations of systemic regenerative therapy using bone marrow stromal cells, when in fact, effective translational success, to date, was always obtained from local transplantation of organ-specific progenitors [e.g., bone marrow orthotopic transplantation (Horwitz et al., 2002); skin regeneration (Green, 1989; De Luca et al., 2006); cornea regeneration (Pellegrini et al., 1997; Rama et al., 2010)]. More importantly, stem cell investigation must be guided by translational demands, in which the primary ambition is to find: cells that can be easily isolated, whether prospectively (based on surface markers), or by plastic adherence of clonal populations; cells that can be clonally expanded *in vitro*; cells that can be locally and not systemically delivered and that will directly engraft or at least support regeneration by establishing a healing microenvironment; or ideally cells that can be regulated *in vivo* by pharmacological stimulation. With that in mind, the recent discovery of the calvarial stem cell niche is well evidenced and presents putative populations that fulfill the basic stem cell criteria, as dictated by translational demands. Whereas *Gli1*^+^ cells are distributed across a large portion of the sutural mesenchyme, expression of *Axin2* and *Prx1* seems to identify more discrete subsets that may or may not be part of a major population (Wilk et al., 2017). Partial overlapping of the proposed markers and slight differences in functional assays should not imply contradictions in calvarial stem cell properties. Rather, the suture mesenchyme most likely comprises a heterogeneous niche in which distinct stem cell subsets can be identified, isolated and characterized with clinical application purposes. Since all recent studies reported *in vitro* clonogenicity of the candidate populations described, a step forward in understanding their biology and potency would be to transplant the expanded colonies to an ectopic site, as the approach addresses the autonomy of the cells in a non-skeletogenic environment.

In humans, while the existence of a calvarial niche residing in the sutural mesenchyme has not been examined, expression of these putative stem cell markers (*Gli1, Axin2*, and *Prx1*) is well predicted based on fetal studies (Homayounfar et al., 2015), analyses of pathogenic skull bones (Coussens et al., 2007) and on our knowledge of signaling pathways crucial in calvarial development. Clearly, the calvarial stem cell niche needs to be further defined in humans if we are to harness these cells for improved skull repair. Isolation and thorough analysis of these cells will then allow us to design new therapies, including introduction of synthetic scaffolds designed to enrich for these cells, or carrying chemotactic cues for to attract these cells to the wound site. Moreover, from the perspective of future clinical translation, two potential autologous stem cell sources are the bone marrow and adipose-derived stem cells. Both of these are heterogenous populations which could be further examined for expression of *Gli1, Axin2*, and *Prx1* positive sub-populations. These markers have not been well characterized in human stem cell populations, although the relevant signaling pathways are implicated in cancer stem cells.

Finally, although the mesenchyme interposing all calvarial bones is predominantly neural crest derived, very little attention was given in the studies reviewed to the distinct embryonic origins of frontal and parietal bones and how this might determine how surrounding stem cells are regulated. Even the mesoderm derived bone is hypothesized to require neural crest (*Sox10*+) lineages residing in the periosteum (Isern et al., 2014). Thus, given the inherent multi-potency of the neural crest cells, osteoblasts derived from that lineage may provide a more plastic milieu than the mesoderm.

## Author contributions

DD, AG, and KL sketched out the initial draft of the review. DD wrote the review and designed the figure. AG and KL revised and edited the review.

### Conflict of interest statement

The authors declare that the research was conducted in the absence of any commercial or financial relationships that could be construed as a potential conflict of interest. The reviewer FW and handling Editor declared their shared affiliation.
